# Towards better understanding of the influence of process parameters in roll compaction/dry granulation on throughput, ribbon microhardness and granule failure load

**DOI:** 10.1016/j.ijpx.2020.100059

**Published:** 2020-11-10

**Authors:** Annika Wilms, Peter Kleinebudde

**Affiliations:** a INVITE GmbH, Drug Delivery Innovation Center (DDIC), Chempark Building W32, 51368 Leverkusen, Germany; b Heinrich Heine University Düsseldorf, Institute of Pharmaceutics and Biopharmaceutics, Universitätsstraße 1, 40225 Düsseldorf, Germany

**Keywords:** Continuous manufacturing, Roll compaction/dry granulation, Microhardness, Granule strength/failure load, Process monitoring, Process analytical technologies

## Abstract

A key quality attribute for solid oral dosage forms is their hardness and ability to withstand breaking or grinding. If the product is to be manufactured continuously, it can be of interest to monitor the hardness of the material at different stages of manufacturing. Using the controlled process parameters of roll compaction/dry granulation specific compaction force, roll speed and gap width, hardness of the resulting ribbons and granules can be predicted. For the first time, in this study two yield variables (corrected torque of the granulation unit and throughput of material) are used to predict the granules failure load. The increase in granule hardness was monitored in-line when the specific compaction force was increased during the compaction process. This opens the way for in-line control of material hardness, and its use for feedback and feedforward control loops for future continuous manufacturing processes.

## Graphical abstract

**Figure:**
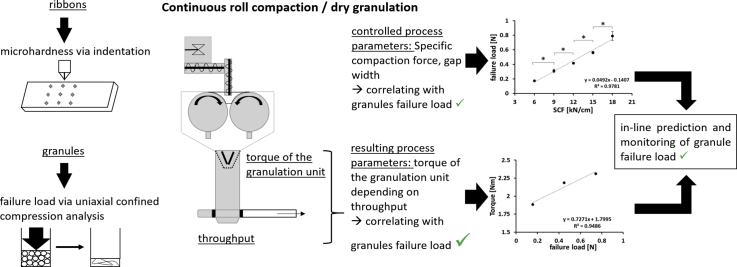

## 1 Introduction

With recent advancements in implementing continuous manufacturing (CM) in pharmaceutical production of solid oral dosage forms there is a need to continuously monitor and control critical quality attributes (CQA) (Chatterjee, 2012; U.S. Food and Drug Administration, 2017; Lee, 2016; Lee et al., 2015; U.S. Food and Drug Administration, 2019). Detailed process understanding is necessary to identify CQAs and their influencing factors. A promising approach to continuous manufacturing, if direct compression is not feasible, is roll compaction/dry granulation (RCDG). It is a well-established, fast, comparably low-cost and, by nature, continuous granulation method, in which powder is compacted to ribbons between two counter rotating rolls and these ribbons are afterwards milled down to granules (Fonteyne et al., 2015; Kleinebudde, 2004; Shlieout et al., 2000). A major CQA for the intermediate ribbon is the ribbon porosity/solid fraction as it impacts the CQAs of the later produced granules, such as granule size distribution (Jaminet and Hess, 1966; Kleinebudde, 2004). In-line methods to determine ribbon solid fraction have been previously published (McAuliffe et al., 2015; Wiedey and Kleinebudde, 2018). However, Gupta et al. showed that microcrystalline cellulose (MCC) ribbons of similar density can vary in hardness (Gupta et al., 2005).

When looking at tablets as the final dosage form, mechanical strength is an essential CQA as it prohibits breaking and grinding. On the other hand, early studies showed that if the mechanical strength is increased dissolution can be impeded (Chowhan, 1979; Kitazawa et al., 1975). In 1985, Jetzer et al. correlated tensile strength with indentation hardness of tablets and showed that the correlation is linear as long as fracture or capping at higher pressure ranges is avoided (Jetzer et al., 1985). This is also backed by more recent work by Patel and Sun, who found that an increase in tablet porosity is linked to a decrease in hardness (Patel and Sun, 2016). Therefore, tablet hardness has similar informational value as the more popular tensile strength and cannot be equated with tablet density (Gupta et al., 2005). In a continuous manufacturing process, based on process understanding it is valuable to track the development of hardness through all intermediates with the aim of achieving optimal tablet hardness/tensile strength.

By definition, hardness is the surface property which quantifies a materials resistance to a permanent shape modification (Broitman, 2017; Walley, 2012). Its relation to further mechanical properties of solid materials (e.g. strength and ductility) is the base for hardness testing as a cheap and fast material quality control method (Broitman, 2017), as mechanical strength is a critical factor for pharmaceutical manufacturing. In RCDG it is valuable information to determine the hardness of ribbons and granules. Reported indentation experiments for pharmaceutical applications are scarce (Hiestand et al., 1971; Jetzer et al., 1985; Leuenberger, 1982; Leuenberger and Rohera, 1986; Rowe, 1976). However, Freitag et al. reported the use of micro-indentation to determine ribbon hardness (Freitag et al., 2004). An increase in the specific compaction force (SCF) during RCDG lead to harder ribbons and, if granules are then tableted, to tablets with decreased tensile strength. Determining granule hardness poses the issue of dealing with agglomerated product of variate particle sizes. Adams et al. first published a confined uniaxial compression test to determine granule strength, and its failure load, which also takes the particle size into consideration (Adams et al., 1994). The method was recently transferred to a modern tablet press (Arndt et al., 2018). Their study proved that increasing SCF leads to granules with increased failure load and tablets with decreased tensile strength.

Studies on correlating the torque of the granulation unit of a roll compactor to ribbon hardness were first published in 2012 (Müller, 2012). A roll compactor including an asymmetrical, oscillating sieve was used to granulate MCC and the clockwise and counter-clockwise torque during granulation was recorded. Hardness of the resulting ribbons was determined using a tablet drill. A correlation between the SCF, the torque values and the drilling forces needed to drill a hole into the ribbon was found. Increasing roll speed increased the torque of the granulation unit while it did not affect the drilling forces. Various aspects of the milling unit (e.g. size of sieve, erosion of the sieve, milling gap) were found to influence the torque. The ribbon hardness was, in retrospective, determined by recording the torque of the granulation unit and interpolation of the correlation between the torque and the ribbon hardness. The throughput was found to have major impact on the torque of the granulation unit, but for further experiments was regarded as a constant as settings were not changed. In RCDG, the throughput can show relevant fluctuations (Wilms et al., 2020), and this should be taken into consideration for in-line determination of CQAs. No experiments were conducted to determine the impact of hardness on the torque independent from the changing throughput as SCF is varied. Furthermore the relation of the torque and hardness to varying process parameters should be analyzed for brittle material and a pharmaceutical formulation as well. Granule hardness was not investigated. For further processing, especially the granule characteristics are of high importance and a method to predict granule characteristics based on ribbon mechanic properties would be valuable.

The same group later focused on the development of using vibration- and acoustic pressure in-line measurements to determine ribbon hardness in-line, which is promising but also bears limitations as the system reacts sensitively to the attachment of the sensors and the surrounding vibration/acoustic environment (Schorr, 2016).

For continuous manufacturing efforts it is of interest to re-evaluate the impact of controlled process parameters (SCF, gap width, roll speed) on microhardness of ribbons and failure load of granules with regards to the torque of the granulation unit (360° rotating conical sieve) for different types of materials. For the first time, the quality parameter of granule failure load was evaluated in conjunction to the torque of the granulation unit and correlations were established for materials with different behavior upon compaction. A correlation was established to predict hardness parameters based on the yield parameters torque of the granulation unit and the throughput of material and the novel principle was tested using real-time granulation data. The study was performed to establish these correlations in order to progress in advanced process control for RCDG.

## 2 Materials and methods

### 2.1 Materials

Dibasic calcium phosphate anhydrate (DCPA, Di-CaFos A150, Chemische Fabrik Budenheim, Germany) was used as brittle model material. Microcrystalline Cellulose (MCC, Vivapur 102, JRS Pharma, Germany) was used as plastic model material.

A formulation was also investigated. It consisted of 25% Diclofenac‑sodium (Amoli Organics Pvt. Ltd., India), 10% crosposvidone (Polyplasdone® XL, Ashland Industries Europe GmbH, Switzerland) and 65% MCC (JRS Pharma, Germany).

### 2.2 Methods

#### 2.2.1 Roll compaction/dry granulation

All materials were processed on a development to small scale manufacturing roll compactor (BRC 25, L.B. Bohle Maschinen + Verfahren GmbH, Germany) that was equipped with a hybrid sealing system and a 360° rotating conical sieve (BTS100, L.B. Bohle Maschinen + Verfahren GmbH, Germany). A 1.5 mm rasp sieve was used for all experiments. Depending on the planned analytics, smooth or knurled rolls were used. The gap width was varied between 1.5 and 2.5, the roll speed between 1 and 6 rpm and the SCF between 2 and 18 kN/cm. The specific process parameters are listed for each experiment in the results section. The throughput of the process was determined using a lab scale balance (CPA5201, Sartorius AG, Germany) that was placed underneath the outlet of the compactor. A collection vessel was placed on the balance and a software (SartoConnect, Sartorius AG, Germany) was used to track the mass every 10 s.

#### 2.2.2 Determination of the torque, the corrected torque and the power of the granulation unit

The torque of the granulation unit was tracked together with the process parameters of the roll compactor in the compactor's software. The parameter is tracked every 10 s in the unit of Nm. It is not the average over 10 s but the as-is value at the tracking time-point. The torque of the granulation unit without material was tracked to determine the idle torque for each impeller speed. Idle torque values are listed in Table 1.

**Table 1:** Idle torque values at different impeller speeds.

Impeller speed [rpm]	Idle torque [Nm]	Idle power consumption [W]
200	1.57 ± 0.02	32.9 ± 0.4
300	1.87 ± 0.02	58.8 ± 0.6
400	2.13 ± 0.01	89.2 ± 0.4

The idle torque values were deducted from the observed torque values to obtain the corrected torque (corr. torque). For results shown in 3.2, 3.3, 3.4, 3.5, 3.6, impeller speeds of 200 rpm were chosen. Therefore, the corrected torque is tracked in these experiments and it is not necessary to convert the corrected torque to power consumption values. To compare different impeller speeds, as needed in Section 3.1, the additional power consumption of the granulation unit (apart from the idle power consumption) was calculated as shown in Eq. (1).

(1) $$\mathrm{power consumpti}on=\mathrm{corr}.\mathrm{torque}*\mathrm{impeller speed}*2*\pi \;\left[W\right]$$

#### 2.2.3 Determination of ribbon microhardness

Ribbon microhardness was determined using a microhardness system (Fischerscope HM2000, Helmut Fischer, Sindelfingen, Germany). The system was equipped with a vickers pyramid indenter which penetrated into ribbons. Penetration depth and indentation load were recorded. A total of 150 data points were recorded in 15 s (10 Hz) for every measurement. The initial 5 s were the loading phase, in which the force increased until 2000 mN were reached. This maximum load of 2000 mN (F_max_) was held for 5 s. Afterwards, for 5 s, the system was unloaded. The maximum penetration depth (h_max_) at maximum indentation load is used to calculate the Martens Hardness (HM) according to Eq. (2) (Jennett, 2007).

(2) $$\mathrm{HM}=F_{\max}/\left(26.43\times {\left(h_{\max}\right)}^{2}\right)\;\left[N/{\mathrm{mm}}^{2}\right]$$

**Fig. 1:**
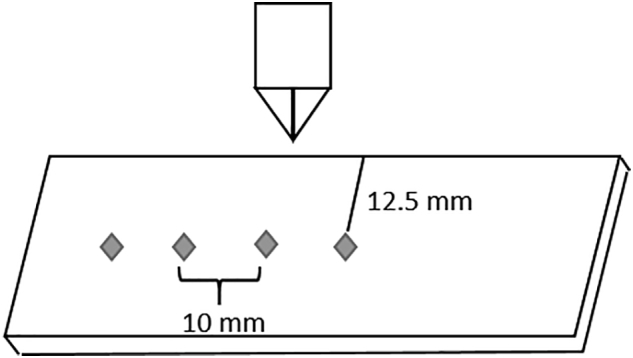
Microindentation experimental procedure.

To allow precise measurements, only ribbons with a smooth surface were used for microhardness determination. A smooth surface allows the indenter to land on an even surface and track penetration depth and indentation load. Ribbons are inhomogeneous concerning their density (Wiedey and Kleinebudde, 2018). Therefore, indentation was only performed in the center of the ribbon (determined with the measurement apparatuses microscope). Measurement spots were 10 mm apart from each other (Fig. 1). Four measurements were performed for each ribbon and averaged.

#### 2.2.4 Determination of granule failure load

Determination of failure load/granule strength was performed based on an uniaxial confined compression analysis. The granules were divided into the following size classes: 125–315 μm, 315–500 μm, 500–800 μm, 800–1250 μm and 1250–1500 μm. Afterwards, they were compressed using a Styl'One Evolution tablet press (Medelpharm S.A.S., France) in a round die of 100 mm^2^ base area and 10 mm height. The upper punch speed was set to 3.5 mm/min and the granule bed was compressed to a final height of 5 mm. Depending on data variability, the test was repeated 3 to 6 times.

Analysis was performed according to Arndt et al. (Arndt et al., 2018) and is based on Eqs. (3), (4) (Adams et al., 1994). The compaction pressure P is calculated from the force and the cross-sectional area of the punch. The natural strain *ϵ* can be obtained using the current and the initial difference in height of the punches. For large values of *ϵ*, the relationship described in Eq. (3) is linear (Adams et al., 1994). The slope and the y-intercept of the linear equation are used to obtain the friction coefficient (*α*) and the cohesive strength (*τ*).

To obtain the individual failure load, the mean value of the cohesive granule strength is multiplied with the particles cross-sectional area. In this case, the cross-sectional area of a sphere with a diameter of the mean value of the sieve class (d_a_) was used for the calculation of each sieve class (see Eq. (4)).

(3) $$\mathrm{lnP}=\ln\left(\frac{\tau }{\alpha }\right)+\alpha \epsilon +\ln\left(1-e^{-\alpha \epsilon }\right)$$

(4) $$F_{\mathrm{calc}}=\frac{\pi d_{a}^{2}}{4}\tau$$

Afterwards, the weighted average failure load for a granule type was calculated by cumulating all size classes' failure loads after multiplying them with their respective relative mass fraction.

#### 2.2.5 Design of experiments

Using MCC as excipient, a 2^3^ full factorial experimental design was conducted to determine the effect of the process parameters SCF, gap width and impeller speed on ribbon microhardness and granule failure load as well as the torque of the granulation unit. Factors and their levels are shown in Table 2. A total of 11 experiments were executed (the center point was tested in threefold). The randomized plan for execution of experiments was given by the software Modde Pro 11 (Umetrics, MKS Data Analytics Solutions, Sweden) and data analysis was also performed using this software.

**Table 2:** Experimental overview.

Factor level	SCF kN/cm	gap width mm	impeller speed rpm
−1	2	1.5	200
+1	6	2.5	400

## 3 Results and discussion

### 3.1 Effect of process parameters on ribbon microhardness, granule failure load, torque of the granulation unit and throughput

**Fig. 2:**
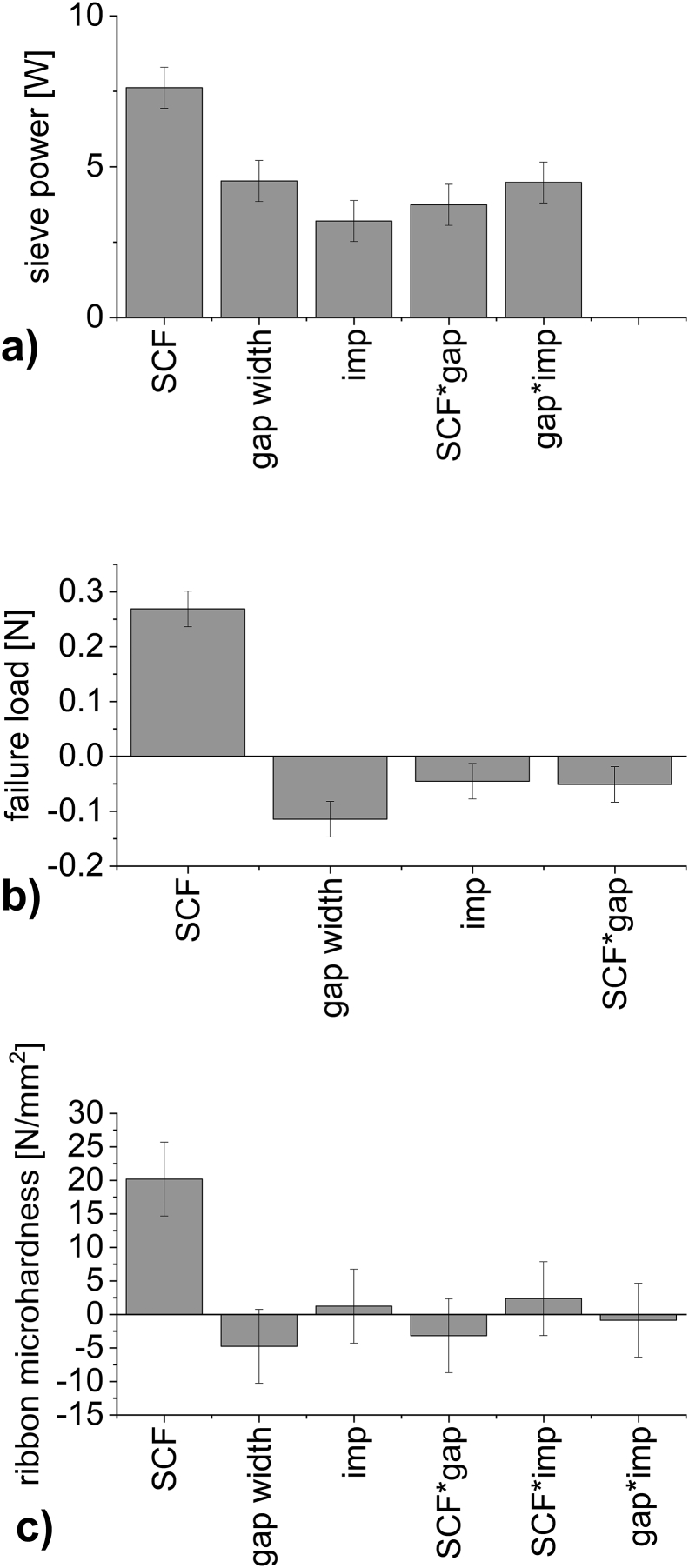
Coefficient plots. Effects on a) sieve power: additional power consumption of the granulation unit, b) failure load and c) ribbon microhardness. Error bars indicating 95% confidence interval (sieve power R^2^: 0.98, Q^2^:0.85., failure load R^2^: 0.99, Q^2^: 0.95, ribbon microhardness R^2^: 0.96, Q^2^:0.74).

**Fig. 3:**
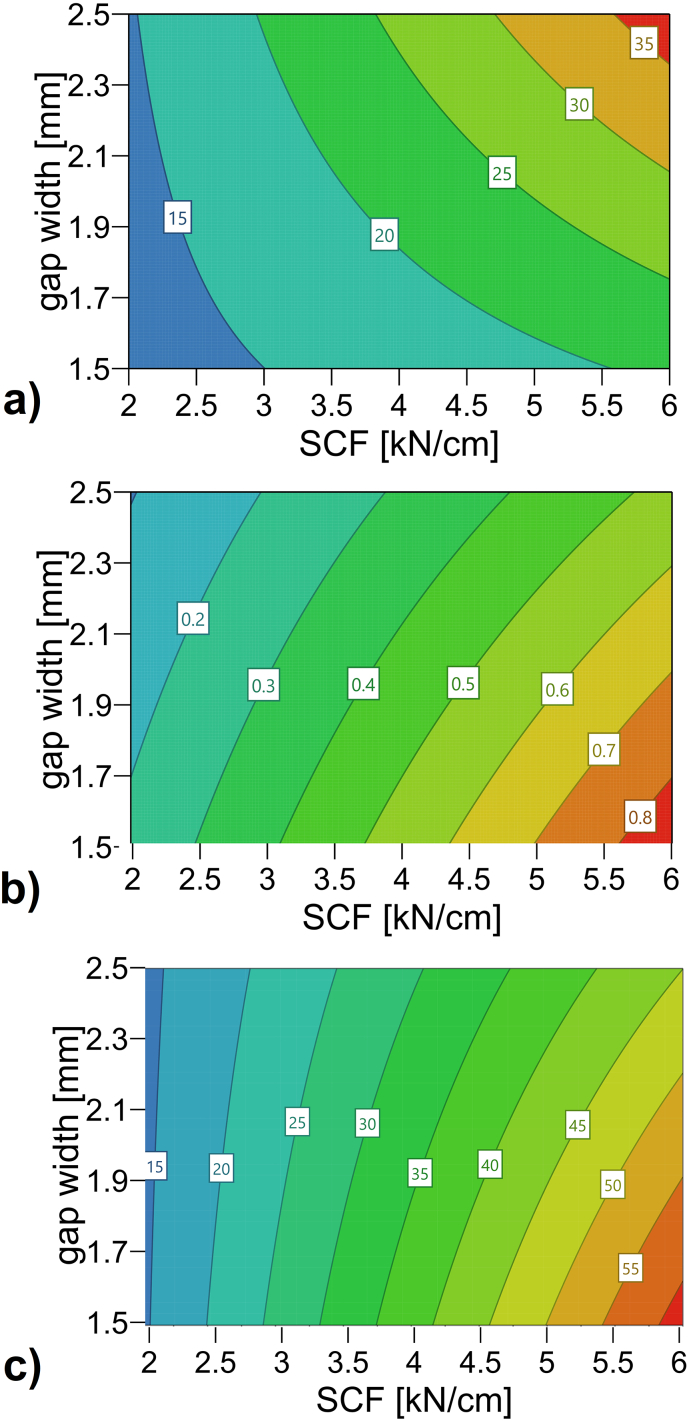
Contour plots. Effects on a) sieve power, b) failure load and c) ribbon microhardness. (sieve power R^2^: 0.98, Q^2^:0.85., failure load R^2^: 0.99, Q^2^: 0.95, ribbon microhardness R^2^: 0.96, Q^2^:0.74).

Table 3 displays the results of the 2^3^ full factorial experiment while Fig. 2 shows the resulting coefficient plots and Fig. 3 exemplary contour plots for varying gap width and SCF.

**Table 3:** Results of the 2^3^ full factorial experimental design, mw ± sd.

Factors			Yield variables				
SCF [kN/cm]	Gap width [mm]	Impeller speed [rpm]	Ribbon microhardness [N/mm^2^]	Granule failure load [N]	Corr. torque of the granulation unit [Nm]	Additional sieve power consumption [W]	Throughput [kg/h]
			*n* = 4	n = 3–6	*n* = 18	n = 18	*n* = 3
2	1.5	200	18.1 ± 4.1	0.27 ± 0.03	0.67 ± 0.09	14.0 ± 1.9	2.92
6	1.5	200	60.8 ± 12.0	0.95 ± 0.10	1.11 ± 0.15	23.3 ± 3.1	4.35
2	2.5	200	17.3 ± 5.0	0.12 ± 0.01	0.42 ± 0.07	8.8 ± 1.5	4.73
6	2.5	200	46.0 ± 7.7	0.60 ± 0.02	1.37 ± 0.41	28.7 ± 8.6	6.84
2	1.5	400	18.2 ± 3.6	0.20 ± 0.02	0.31 ± 0.06	13.0 ± 2.5	2.82
6	1.5	400	69.0 ± 11.6	0.80 ± 0.05	0.46 ± 0.14	19.3 ± 5.9	4.05
2	2.5	400	12.7 ± 1.5	0.09 ± 0.01	0.51 ± 0.06	21.4 ± 2.5	4.89
6	2.5	400	52.0 ± 4.7	0.48 ± 0.03	1.12 ± 0.22	46.9 ± 9.2	6.16
4	2	300	36.2 ± 2.5	0.43 ± 0.01	0.62 ± 0.16	19.5 ± 5.0	4.99
4	2	300	30.4 ± 2.8	0.41 ± 0.02	0.69 ± 0.09	21.7 ± 2.8	5.06
4	2	300	26.1 ± 9.7	0.37 ± 0.02	0.62 ± 0.16	19.5 ± 5.0	5.68

Increasing the SCF lead to a significant increase in failure load of the granules, the ribbon microhardness as well as the additional power consumption of the granulation unit. For all output parameters the SCF showed the largest impact. As it is the dominant process parameter in RCDG this is not surprising and in-line with published research (Arndt et al., 2018; Freitag et al., 2004; Müller, 2012). The increased ribbon hardness could lead to the significant increase the SCF exerted on the additional power consumption of the granulation unit. However, the impact of increased throughput at increased SCF has to be kept in mind. This can be seen as the impact of the process parameter gap width is taken into consideration. At constant SCF but increased gap width, the SCF is exerted on more material. Therefore, the material itself shows decreased granule failure load (Fig. 2 b) and 3 b)). However, while the hardness decreases, the additional power consumption of the granulation unit increases significantly (Fig. 2 a) and 3 a)). This can be reasoned in the increased throughput at larger gap widths. To no surprise, the interaction of increased SCF and increased gap width increases the additional power consumption of the granulation unit as well (Fig. 2 a) and 3 a)).

Ribbon microhardness significantly increases at increasing SCF (Fig. 2 c) and 3 c)). The SCF is the only process parameter that has a significant impact on ribbon microhardness (Fig. 2 c)). This can be due to the fact that ribbon microhardness characterizes the surface of the ribbon. The surface is also the part of the ribbon on which the SCF is exerted on. Effects of increasing the gap width on ribbon microhardness are not significant in contrast to results of granule failure load.

By nature, adjusting the impeller speed affects the additional power consumption of the granulation unit (Fig. 2 a), Eq. (1)). At an increased impeller speed, granules show a smaller granule size (Mangal and Kleinebudde, 2018) and a slight decrease in failure load (Fig. 2b)). Since the impeller speed cannot influence the ribbon microhardness, this factor and all its interactions show non-significant effects close to zero on ribbon microhardness (Fig. 2c)).

As expected, the results of the experiments show that hardness of the ribbons and failure load of the granules react similarly to changes of SCF. The torque of the granulation unit reacts to the changes in process parameters as well but not in the same way as the hardness does. Supposedly, this is based on the effect of the throughput and it has to be analyzed in detail, whether a change in hardness itself has an impact on the torque of the granulation unit. As an increase in SCF will increase the failure load of the granules (Arndt et al., 2018) and similarly the ribbon microhardness (Freitag et al., 2004), only the granules failure load was used for a more detailed analysis of impact on the additional power consumption of the granulation unit. Its behavior can be correlated to the behavior we would expect from ribbon microhardness. In the more detailed analysis, the sieve impeller speed will be kept constant at 200 rpm, therefore it is not necessary to calculate the additional power consumption of the granulation unit. The corrected torque of the granulation unit bears all necessary information (see Eq. (1)).

### 3.2 Influence of SCF on torque of the granulation unit and granule failure load

**Fig. 4:**
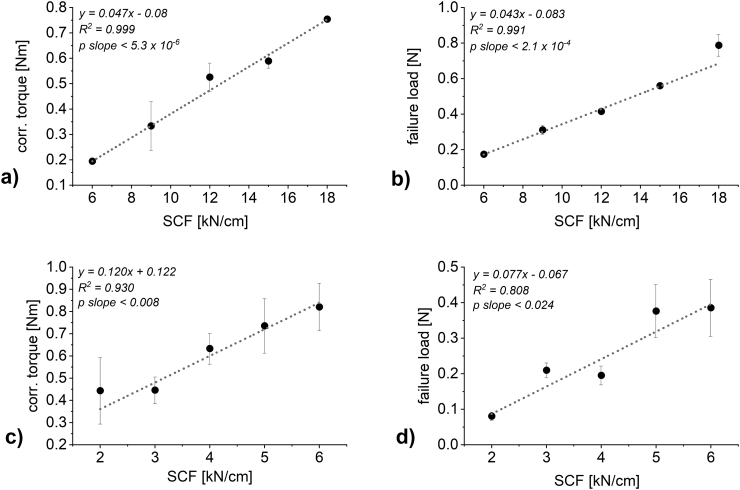
Plot of SCF against a) and c) the torque of the granulation unit (*n* = 3–7; mean ± sd) and b) and d) the failure load of the resulting granules (*n* = 5; mean ± sd). a) and b) DCPA c) and d) MCC.

Fig. 4 shows the impact of SCF on the torque of the granulation unit and the failure load of the resulting granules at otherwise constant controlled process parameters (2 mm gap width, 3 rpm roll speed for DCPA and 1 rpm roll speed for MCC). Similarly to the results in Fig. 2, increasing the SCF lead to an increase in the corrected torque of the granulation unit (Fig. 4 a) and c)) and failure load of the resulting granules (Fig. 4 b) and d)). In both cases a linear correlation describes the applied SCF and the resulting torque of the granulation unit (Fig. 4 a) and c)).

Correlation of SCF and failure load (Fig. 4 b) and d)) works well for DCPA but shows a lower coefficient of determination R^2^ of 0.808 for MCC. Obtaining reproducible failure load values for high SCF for MCC was challenging, as reproducible volumetric filling of the die is more difficult, if the granule sizes are large. At 5 and 6 kN/cm, a large quantity of material was in size classes of 850–1200 μm and 1200–1500 μm. The impact of these difficulties was larger, if more material was produced at these sizes as the weighted average was calculated (see 2.2.4. Determination of granule failure load). Based on these difficulties, linear correlation results for MCC are acceptable.

It is therefore possible to predict the failure load of the granules based on linear correlation when looking at the SCF at constant gap width and roll speed. The linear increase of the torque of the granulation unit has to be further investigated, as the increase based on increased hardness and increased throughput have to be differentiated.

### 3.3 Impact of SCF and roll speed on throughput

**Fig. 5:**
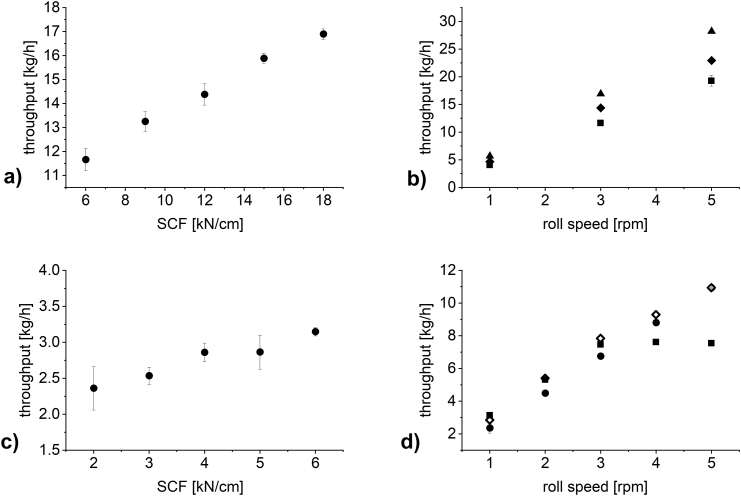
Plots of a) and c) SCF against throughput, (n = 3–6; mean ± sd) and b) and d) roll speed against throughput for 2 kN/cm (unfilled circle), 4 kN/cm (unfilled square), 6 kN/cm (square), 12 kN/cm (circle) and 18 kN/cm (triangle) (n = 3–6; mean ± sd). a) and b) DCPA, c) and d) MCC. Gap width in all experiments: 2.0 mm.

Fig. 5 shows the impact of the SCF and roll speed on the throughput of material for DCPA (Fig. 5 a) and b)) and MCC (Fig. 5 c) and d)). As expected, the throughput increased at increasing SCF and, for a constant SCF, increased at increasing roll speed. Compacting MCC at 6 kN/cm and increasing the roll speed (Fig. 5 d), squares) did not lead to increasing throughput at 4 rpm and above. There was a decrease of throughput at 5 rpm compared to 4 rpm. This can be reasoned, as during production, the milling unit filled up with material when compacting at 6 kN/cm and above 3 rpm roll speed. Less material could be milled down than was produced leading to an accumulation of material in the mill and a stagnant throughput. In all other cases all material could be milled down.

### 3.4 Impact of roll speed on the torque of the granulation unit and granule failure load

As mentioned in Section 3.2., the impact of throughput and hardness on the torque of the granulation unit have to be differentiated.

**Fig. 6:**
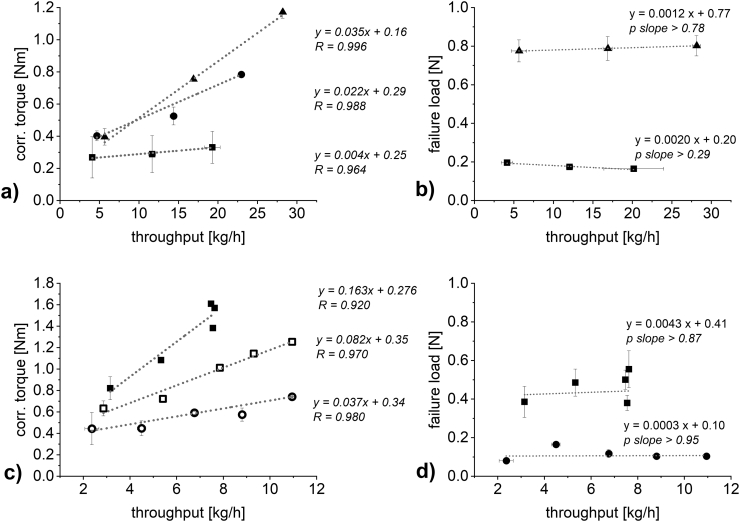
Plot of throughput against a) and c) the torque of the granulation unit (*n* = 1–6; mean ± sd) b) and d) the failure load of the resulting granules (n = 3–5; mean ± sd). 2 kN/cm (unfilled circle), 4 kN/cm (unfilled square), 6 kN/cm (square), 12 kN/cm (circle) and 18 kN/cm (triangle). a) and b) DCPA and c) and d) MCC. Gap width in all experiments: 2.0 mm.

At three SCFs, the speed of the compaction rolls was varied (1, 3 and 5 rpm for DCPA and 1, 2, 3, 4 and 5 rpm for MCC) to vary the throughput of material (Fig. 6 a) and c)). In all cases, an increase in throughput lead to an increase in torque of the granulation unit. The plot is an interaction plot of throughput on the torque of the granulation unit at varying SCF. For both materials, an increase in SCF resulted in an increased slope of the throughput – torque correlation. Therefore, the impact of the throughput is dependent on the applied SCF. More specifically, at higher SCF the impact of throughput on the resulting torque of the granulation unit will increase. This can be reasoned in the increasing hardness at increasing SCF (Fig. 4 b) and d)).

Increasing roll speed reduces the dwell time in the compaction zone. Therefore, it had to be tested, whether increasing the roll speed might decrease the hardness of the resulting granules. For both materials, the failure load of granules produced at two SCFs (6 and 18 kN/cm for DCPA and 2 and 6 kN/cm for MCC, Fig. 6 b) and d)) were tested at varying throughputs. Concerning DCPA, the probability of the slope being zero is above 79% at 18 kN/cm and above 29% at 6 kN/cm. The low percentage at 6 kN/cm is based on the low standard deviations obtained in failure load measurements. No relevant trend in granule hardness can be found for both of the SCF. Detailed values are included in the supplementary material (see Table S1 for raw data DCPA and Table S2 for raw data MCC). For MCC, obtaining reproducible failure load values at 6 kN/cm was challenging and thus, relevant standard deviations were obtained. This can be reasoned in the previously discussed lacking impact on throughput of increasing the roll speed above 4 rpm (see Section 3.3.) resulting in a cluster of values located slightly below 8 kg/h. Furthermore, as discussed in Section 3.2., it was difficult to obtain reproducible results of failure load due to die filling issues. However, there is no visible trend to decreasing hardness at increased roll speeds for both SCF. The probability of the slope equaling zero is above 87% and above 95% for 6 kN/cm and 2 kN/cm respectively. Therefore, the hardness values plotted in Fig. 4 b) and d) are the experimentally determined values for a certain SCF regardless of the roll speed (see Tables S1 and S2).

**Fig. 7:**
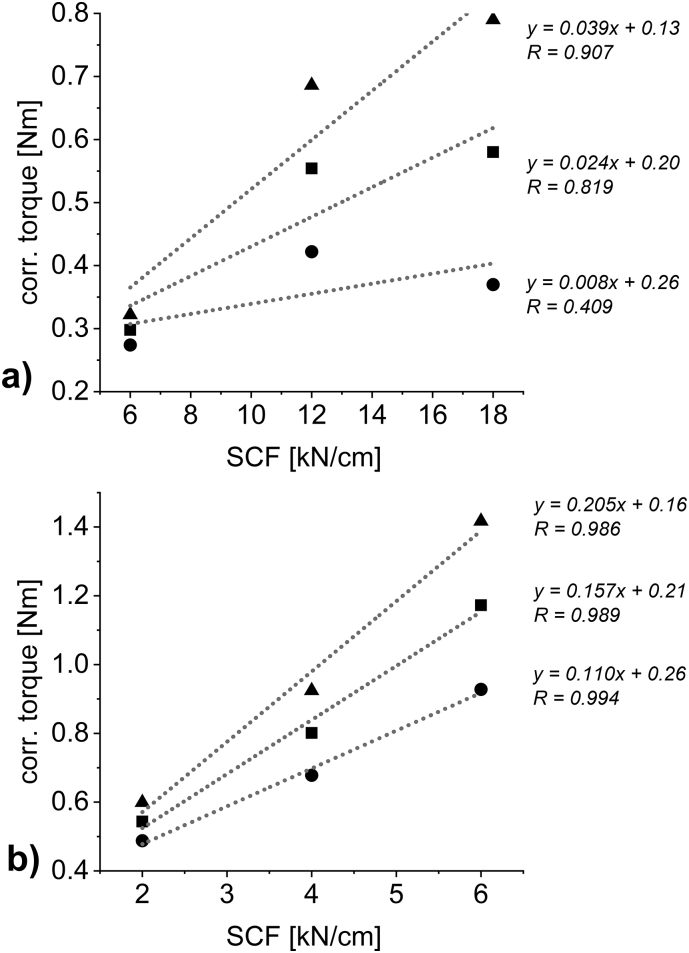
Interaction plot of SCF against the torque of the granulation unit at varying throughputs for a) DCPA; 6 kg/h (circle); 12 kg/h (square); 18 kg/h (triangle) and b) MCC; 4.0 kg/h (circle); 5.5 kg/h (square); 7.0 kg/h (triangle).

Fig. 6 a) and c) show the throughput – corr. torque of the granulation unit plots including their linear correlation and correlation coefficients R. To evaluate the sole effect of SCF on the torque of the granulation unit, the linear correlations were used to calculate torque values at specific throughputs. The resulting plot (Fig. 7) is the interaction plot of SCF on the corrected torque of the granulation unit depending on different throughputs. In all cases, increasing the SCF at constant throughputs resulted in higher corrected torque values of the granulation unit. While for MCC linear correlations show R-values of above 0.98 (Fig. 7 b)), this is not the case for DCPA (Fig. 7 a)). The data points seem as if, for the brittle material, at increasing SCF the torque values could flatten while hardness still increases linearly (Fig. 4 b)). This non-linearity is based on data shown in Fig. 6 a). The torque values of the material produced at 12 kN/cm is not located centered between 6 and 18 kN/cm.

For both materials, the slope increases at increasing throughput proving that the impact of SCF is more profound at higher throughputs and vice versa. This plot confirms that independent from throughput, the SCF, and thus increasing hardness of the material, has impact on the torque of the granulation unit.

It can be concluded that increasing the throughput by increasing compaction roll speed will not affect the hardness of the resulting granules. Therefore, material of similar hardness will generate different torque values based on the current throughput. These torque values increase steeper for harder materials. The effect of the hardness itself on the torque of the granulation unit was proven. If the throughput of material is known, the hardness should therefore be predictable by using the corrected torque of the granulation unit.

### 3.5 Prediction of hardness based on resulting process parameters

The prediction of granule hardness was tested by using data obtained by compacting DCPA at 15 kN/cm and MCC at 5 kN/cm (see Table 4, Fig. 4 and Tables S1 and S2 in the supplementary material). Based on the results discussed in 3.2, 3.4, two predictions can be made.

**Table 4:** SCF, torque of the granulation unit and throughput values for specific data points.

SCF [kN/cm]	Torque of the granulation unit [Nm]	Throughput [kg/h]	Failure load [N] – pred.	Failure load [N] – calc.	Failure load [N] – meas.
*DCPA*					
15	2.163	15.89	0.55	0.56	0.56

*MCC*					
5	2.310	2.87	0.39	0.32	0.38

Firstly, based on the linear correlation of SCF and failure load (Fig. 4 b) and d)) the failure load at a certain SCF can be calculated. This is shown in Table 4 as “failure load [N] – calc”. Only the applied SCF is necessary for this calculation since it was determined that roll speed does not influence the resulting granules failure load (Fig. 6 b) and d)).

**Fig. 8:**
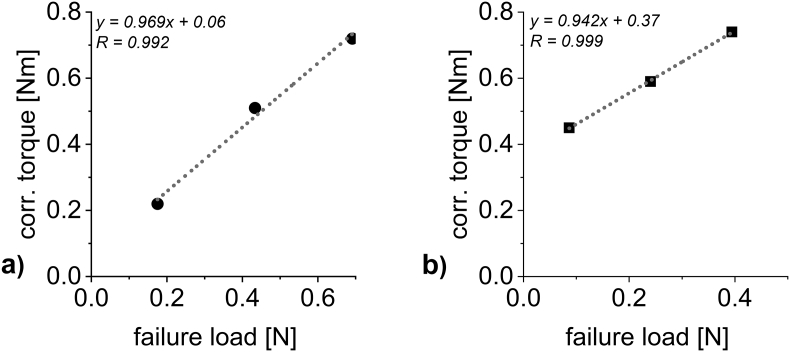
Plots of failure load against the torque of the granulation unit for throughputs of a) DCPA, 15.89 kg/h and b) MCC, 2.87 kg/h.

Secondly, a prediction of granule failure load based on current throughput and corrected torque can be made. The correlations shown in Fig. 6 a) and c) can be used to determine the torque values expected for the current throughput at three different SCF. Since these SCF linearly translate to granule failure load values (Fig. 4 b) and d)), a granule failure load – corrected torque of the granulation unit plot can be derived (Fig. 8). Using the average corrected torque that was registered during granulation, a predicted failure load value of the resulting granules can then be calculated using the linear correlations shown in Fig. 8. The result is listed as “failure load [N] – pred.” In Table 4.

These values can then be compared to the failure load measured (Table 4, “failure load [N] – meas.”, data points plotted in Fig. 4 b) and d)).

The variation between the measured and the calculated failure load can also be seen in Fig. 4 b) and d). They are described by the coefficient of variation R^2^ (0.991 for DCPA and 0.808 for MCC). The value for DCPA (0.56 N), lies almost perfectly on the linear correlation plot whereas, in an overall less convincing correlation for MCC based on strong fluctuations, the calculated failure load for 5 kN/cm deviates stronger from the measured value (Fig. 4 d)). Therefore, the calculated and measured failure load for DCPA compacted at 15 kN/cm are both 0.56 N while they differ (0.32 N to 0.38 N) for MCC.

The predicted failure load values deviate from the measured and calculated ones. For DCPA, the predicted failure load is slightly below the measured failure load (0.55 N to 0.56 N), however this difference cannot be regarded as relevant. In conclusion, both methods to determine the granule failure load in-line (based on SCF only and based on corrected torque and throughput) can be regarded as equally suitable. For MCC, the prediction of granule hardness based on the corrected torque and the throughput outshine the prediction based on SCF alone. With only a slight deviation to the measured value (0.39 N to 0.38 N), there is no relevant discrepancy in granule failure load using the real-time prediction based on resulting process parameters. Therefore, the method has proven usable for real-time hardness prediction.

**Fig. 9:**
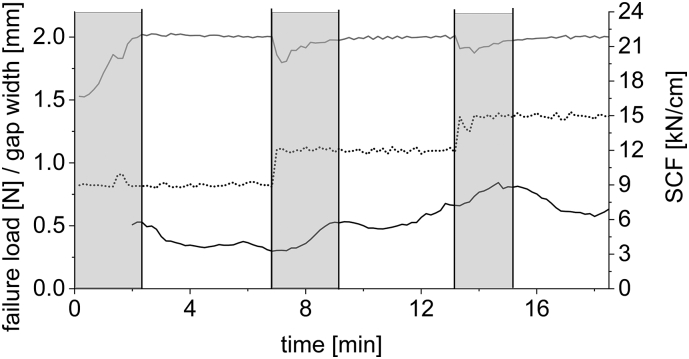
Plot of failure load (solid black), gap width (solid gray) and SCF (dotted black) against time. Gray coloring highlights time periods of instable process conditions. DCPA as excipient. Failure load moving average over 2 min. Gap width and SCF values every 10 s. Real-time data, *n* = 1.

Fig. 9 displays results of an experiment compacting DCPA. The process parameters SCF and gap width are plotted against time. Furthermore, the predicted failure load is plotted in black. It was predicted using real-time throughput and corrected torque data. The throughput was determined every minute and a linear correlation between failure load and torque was determined for every throughput. The current corrected torque values were then used to determine the predicted failure load. The moving average over 2 min was plotted. Gray areas highlight process parameters that were not in equilibrium, as a change in SCF lead to a necessary adjustment in feeding screw speed and an instability in gap width. As shown in Fig. 2 c), both of these process parameters have significant effects on the granules failure load. Therefore, for each SCF the failure load values predicted in process equilibrium should be evaluated. It can be seen that the failure load, despite being calculated by using only the current throughput and torque values, reacted to changes in process parameters. An increase in SCF lead to an increase in the granules failure load.

### 3.6 Relevance for pharmaceutical manufacturing – how do formulations behave?

To test whether the dependencies of torque, throughput and SCF are also valid for a mixture of API and excipients, a formulation was prepared and granulated. Raw data is listed in Table S3 in the supplementary material.

**Fig. 10:**
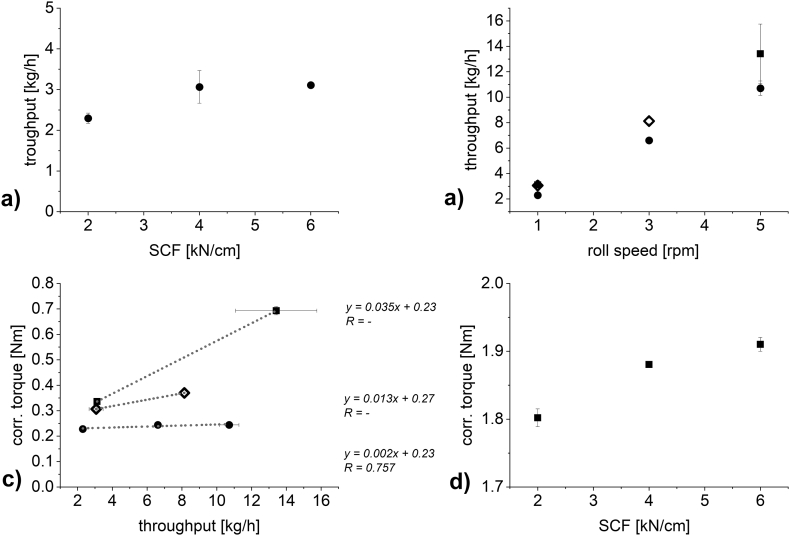
Plots of a) SCF against throughput b) roll speed against throughput at 2 kN/cm (circle), 4 kN/cm (horizontal line), 6 kN/cm (square) c) throughput against corrected torque of the granulation unit d) SCF against corrected torque of the granulation unit. n = 1–3; mean ± sd.

Fig. 10 shows plots for the formulation comparable to those previously shown for pure excipients. It can be seen that the formulation behaves similarly to the pure excipients as trends that were reported previously are found for the formulation as well.

**Fig. 11:**
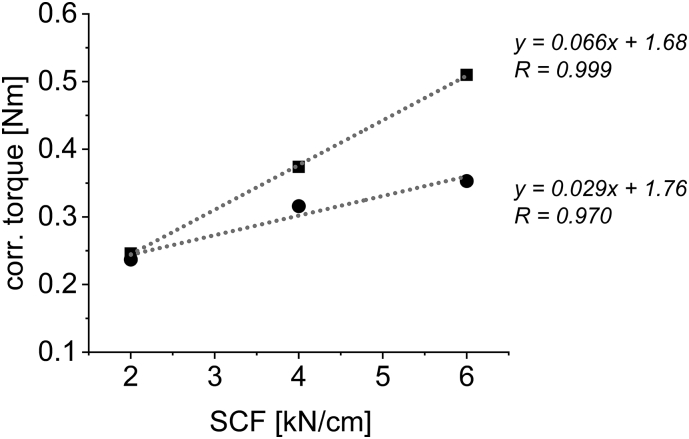
Plot of SCF against the interpolated corrected torque of the granulation unit at 3.5 kg/h (circle) and 8 kg/h (square).

Analogous to Fig. 7 it is possible to plot the correlation of SCF and the torque of the granulation unit independent from throughput (Fig. 11). Good linear correlations are obtained for the two throughputs furthest apart that could be compared using interpolation.

It can be concluded that the behavior of the torque of the granulation unit while processing a formulation is similar to pure excipients.

## 4 Conclusion

Linear correlations of SCF and failure load were established for two materials. It was proven that failure load is independent from the chosen roll speed. Based on the torque of the granulation unit that were measured for different SCF at varying throughputs, a correlation was established that allows to evaluate the impact of the material hardness on the torque of the granulation unit for a specific throughput at constant impeller speed. This correlation was used to predict the granule hardness completely based on process parameters. Therefore, the expected failure load of granules produced with controlled process parameters e.g. SCF and gap width can be compared to the in-line predicted failure load based on resulting process parameters (throughput and the corrected torque of the granulation unit). This opens the possibility of a feedback control loop based on granule failure load controlling the SCF.

Furthermore, if the current granule failure load can be predicted, the optimal compression pressure to obtain tablets of a certain tensile strength could be calculated. This could lead to an in-line determination of compression pressure that can be incorporated into a feedforward control loop. This would allow varying the process parameter of compression pressure in real-time in order to obtain in-specification tablets during the whole production run. More work and validation of the shown correlations is required in order to implement these control loops.

## Funding

This research did not receive any specific grant from funding agencies in the public, commercial, or not-for-profit sectors. This work was supported by the Drug Delivery Innovation Center (DDIC), INVITE GmbH, Leverkusen.

## Declaration of Competing Interest

The authors declare that they have no known competing financial interests or personal relationships that could have appeared to influence the work reported in this paper.

## Appendix A Supplementary data

Supplementary material: Table S1. Results of throughput, corrected torque and failure load values for DCPA; Table S2. Results of throughput, corrected torque and failure load values for MCC; Table S3. Results of throughput and corrected torque for the formulation Image 1
