# Supplementary material for: Towards better understanding of the influence of process parameters in roll compaction/dry granulation on throughput, ribbon microhardness and granule failure load
Source: Int J Pharm X. 2020 Nov 10;2:100059. doi: 10.1016/j.ijpx.2020.100059 (PMC7679258; doi:10.1016/j.ijpx.2020.100059)
Supplement: Supplementary file 1 — Supplementary material: Table S1. Results of throughput, corrected torque and failure load values for DCPA; Table S2. Results of throughput, corrected torque and failure load values for MCC; Table S3. Results of throughput and corrected torque for the formulation [file mmc1.docx]

**Supplementary material**

***Table S1. Results of throughput, corrected torque and failure load values for DCPA***

| SCF [kN/cm] | Roll speed [rpm] | throughput  [kg/h] | corr. torque  [Nm] | failure load  [N] |
| --- | --- | --- | --- | --- |
|  |  | n = 3 - 6 | n = 3 - 6 | n = 5 |
| 6 | **1** | 4.1 ± 0.7 | 0.13 ± 0.02 | 0.20 ± 0.01 |
| 6 | **3** | 11. ± 0.5 | 0.29 ± 0.12 | 0.17 ± 0.01 |
| 6 | **5** | 19.3 ± 1.0 | 0.34 ± 0.10 | 0.17 ± 0.01 |
|  |  |  |  |  |
| 12 | **1** | 4.7 ± 0.2 | 0.41 ± 0.03 | - |
| 12 | **3** | 14.4 ± 0.4 | 0.53 ± 0.06 | - |
| 12 | **5** | 23.0 ± 0.1 | 0.79 ± 0.01 | - |
|  |  |  |  |  |
| 18 | **1** | 5.7 ± 1.0 | 0.40 ± 0.05 | 0.78 ± 0.06 |
| 18 | **3** | 16.9 ± 0.6 | 0.76 ± 0.01 | 0.79 ± 0.06 |
| 18 | **5** | 28.2 ± 0.5 | 1.18 ± 0.04 | 0.80 ± 0.05 |
|  |  |  |  |  |
| 9 | **3** | 13.3 ± 0.4 | 0.34 ± 0.10 | 0.31 ± 0.03 |
| 15 | **3** | 15.9 ± 0.5 | 0.59 ± 0.08 | 0.56 ± 0.02 |

***Table S2. Results of throughput, corrected torque and failure load values for MCC***

| SCF [kN/cm] | Roll speed [rpm] | throughput  [kg/h] | corr. torque  [Nm] | failure load  [N] |
| --- | --- | --- | --- | --- |
|  |  | n = 1-3 | n = 1- 3 | n = 3 - 5 |
| 2 | **1** | 2.4 ± 0.3 | 0.45 ± 0.15 | 0.08 ± 0.01 |
| 2 | **2** | 4.5 ± 0.2 | 0.45 ± 0.07 | 0.17 ± 0.02 |
| 2 | **3** | 6.8 ± 0.1 | 0.60 ± 0.01 | 0.12 ± 0.02 |
| 2 | **4** | 8.8 ± 0.2 | 0.58 ± 0.06 | 0.10 ± 0.01 |
| 2 | **5** | 11.0 ± 0.0 | 0.75 ± 0.00 | 0.10 ± 0.01 |
|  |  |  |  |  |
| 4 | **1** | 2.9 ± 0.1 | 0.64 ± 0.07 |  |
| 4 | **2** | 5.4 ± 0.10 | 0.73 |  |
| 4 | **3** | 7.9 | 1.02 |  |
| 4 | **4** | 9.3 | 1.15 |  |
| 4 | **5** | 11.0 | 1.25 |  |
|  |  |  |  |  |
| 6 | **1** | 3.2 ± 0.1 | 0.83 ± 0.11 | 0.39 ± 0.08 |
| 6 | **2** | 5.3 | 1.09 | 0.49 ± 0.07 |
| 6 | **3** | 7.5 | 1.61 | 0.50 ± 0.05 |
| 6 | **4** | 7.6 | 1.57 | 0.56 ± 0.10 |
| 6 | **5** | 7.6 | 1.39 | 0.38 ± 0.04 |
|  |  |  |  |  |
| 3 | **1** | 2.5 ± 0.1 | 0.45 ± 0.06 | 0.21 ± 0.02 |
| 5 | **1** | 2.9 ± 0.2 | 0.74 ± 0.12 | 0.38 ± 0.08 |

***Table S3. Results of throughput and corrected torque for the formulation***

| SCF [kN/cm] | Roll speed [rpm] | throughput  [kg/h] | corr. torque  [Nm] |
| --- | --- | --- | --- |
|  |  | n = 2-4 | n = 3- 4 |
| 2 | **1** | 2.3 ± 0.1 | 0.23 ± 0.01 |
| 2 | **3** | 6.6 ± 0.18 | 0.25 ± 0.01 |
| 2 | **5** | 10.7 ± 0.6 | 0.25 ± 0.01 |
|  |  |  |  |
| 4 | **1** | 3.1 ± 0.4 | 0.31 ± 0.00 |
| 4 | **3** | 8.1 ± 0.2 | 0.37 ± 0.02 |
|  |  |  |  |
| 6 | **1** | 3.1 ± 0.1 | 0.34 ± 0.01 |
| 6 | **5** | 13.4 ± 2.3 | 0.70 ± 0.02 |
